# Rapid weight reduction does not impair athletic performance of Taekwondo athletes – A pilot study

**DOI:** 10.1371/journal.pone.0196568

**Published:** 2018-04-26

**Authors:** Woo-Hwi Yang, Oliver Heine, Marijke Grau

**Affiliations:** 1 Department of Molecular and Cellular Sport Medicine, Institute of Cardiovascular Research and Sport Medicine, German Sport University Cologne, Cologne, Germany; 2 Olympic Training Centre Rhineland, Cologne, Germany; Medical University of Vienna, AUSTRIA

## Abstract

In combat sports such as taekwondo (TKD), athletes rapidly reduce body weight to achieve a desired weight category. Competition takes place 16–24 h after weigh-in and thus, the recovery time is an important factor for competition performance. The purpose of this study was to investigate the impact of rapid weight reduction (RWR) on athletic performance and associated hemorheological properties considering relevant recovery time. Five male TKD athletes reduced body weight by 5% within 3½ days. A simulated competition day (SCD) was carried out after a 16 h recovery period. Parameters were measured before RWR, at weigh-in and before and after three TKD simulation matches (SMs) at SCD. Same set-up was conducted but without RWR as control. Basal blood parameters, red blood cells (RBC) deformability and aggregation, serum glucose and fibrinogen were determined. During SMs, heart rate (HR_peak_, HR_mean_), oxygen uptake (VO_2peak_, VO_2mean_), peak lactate (Peak La^-^), difference of lactate (ΔLa) and energy systems (anaerobic-alactic, -lactic and aerobic) were analyzed. Basal blood parameters remained unaltered during the interventions. RBC deformability was reduced and aggregation was increased after RWR but values returned to baseline after recovery and were not affected by the SMs. Glucose level was not affected by the interventions. Kick frequency in SMs was higher after RWR which might be responsible for higher HR_peak_, VO_2peak_, VO_2mean_, Peak La^-^, ΔLa^-^ and aerobic demand. The 16 h recovery is sufficient to regenerate measured physiological and hemorheological parameters. TKD-specific performance was not negatively affected during SMs after RWR.

## Introduction

The combat sport Taekwondo (TKD) is an Olympic discipline since the 2000 Olympic Games in Sydney/Australia. In TKD, all fights–from qualification to finals–take place in one day [1]. TKD athletes compete in a designated weight category which is controlled by official weigh-in. The weigh-in takes place in the afternoon prior to the competition day [1] and TKD athletes aim to fight in the next lighter weight category to compete with smaller and lighter opponents [2–4]. In this regard, TKD athletes rapidly reduce their body weight in a range of 2–10% [2, 4–10]. Rapid weight reduction (RWR) is achieved by losing body water during a short-term period. Strategies to reduce body weight include food restriction such as fasting and dehydration, increased training intensity and volume with thermal clothing and sauna sessions [4, 5, 11]. Previous studies indicated that RWR increases oxidative stress, leads to an imbalance of electrolytes and hormones, decreases glycogen storage, changes in blood flow, and decreases plasma volume [12–18]. Further, a recent study showed that 5% RWR decreases red blood cell (RBC) deformability and increases RBC aggregation [8].

Deformability and aggregation of RBC are important factors ensuring oxygen (O_2_) transport into the working muscle cell which is associated to athletes´ performance [8, 19–21]. RBC must deform to pass through capillaries and to supply O_2_ to the working muscle and organs. Blood fluidity, cell surface area-to-volume ratio and physiological properties of the membrane and cytoskeleton were shown to affect RBC deformability [20, 21]. RBC aggregation indicated by face-to-face rouleaux formation upon changes in plasma protein concentration [22]. Increased plasma fibrinogen concentration is believed to increase RBC aggregation. Fibrinogen affects all aspects of RBC aggregation such as aggregate size, stress, viscosity of red cell suspensions and RBC sedimentation [23–25]. It is believed that the recovery time between weigh-in and competition represents a key factor determining the performance during competition after RWR. In TKD, judo and wrestling, 16–24 h lie in-between weigh-in and competition [3, 26]. This period is used to recover body water and carbohydrate storage and to manage possible gastrointestinal distress after RWR [4, 8, 26, 27].

Analyzing recent studies implies a difference between physical performance in laboratory tests and athletic performance in real competition after RWR. Strength, power, peak oxygen uptake (VO_2peak_), and anaerobic performance measured in a Wingate test were not affected by RWR [11, 28–31]. These appear to be controversially discussed because of different phases and percentages of RWR and diverse measurements of athletic performances [7, 11, 28, 29]. The true competitive benefit that a combat athlete may have by facing smaller opponents may overcome small harmful effects on physical capacities such as strength and aerobic or anaerobic fitness. Actually, no study assessing physical capacities in laboratory settings will ever be able to fully explain how RWR affects actual competitive performance [32].

The outcomes of aerobic and anaerobic performance tests highly depend on the time period between weigh-in and performance test [10]. Artioli et al. [10] suggest that several studies allowed no or very short recovery periods (<1h) which indicated negative effects of RWR on physical performance, but when the recovery periods are longer (>3h), RWR typically does not impair physical performance.

From aforementioned studies it is not clear if and how RWR affects TKD-specific performance but also blood flow related parameters such as deformability and aggregation after a practically relevant recovery time. Therefore, the aim of the present pilot study was to examine the changes of different energy demands, physiological and hemorheological responses after a 3½ day RWR, including 16 h recovery period and during multiple simulation matches on the simulated competition day in TKD athletes.

## Materials and methods

### Ethical approval

The study was approved by the ethics committee of the German Sport University Cologne (No. 53/2015). The applied protocols align with the Declaration of Helsinki. All participants signed an informed consent form.

### Subjects and study design

Five male regional level taekwondo athletes (black or red and black striped belt; degree: at least 1^st^ Dan) who belong to the light weight categories (range: -54 up to -68 kg) participated in this study. The anthropometric data of the participants were as follows (Mean ± SD): age: 17.40 ± 1.67 years, body weight: 57.77 ± 7.04 kg, height: 170.30 ± 4.91 cm. Average endurance training volume was between 10–12 h per week and all participants exhibited individual experiences in body weight loss. The athletes did not take any medication during all procedure and did without alcohol and nicotine consumption for at least 24 h before the study.

The study consisted of two phases, a control phase without body weight reduction and a RWR phase in which the participants were told to reduce body weight by 5% during 3½ days according to previous studies [4–8]. Due to study organization to display real weigh-in time, the duration of RWR was modified (3½ days instead of 4 days). Weigh-in was scheduled at the fourth day of RWR [4, 7, 8]. During RWR, weight loss was conducted by individual methods of athletes and included fasting, decreased fluid intake, dehydration trough sauna sessions, increased training intensity/volume. Nutritional intake and activity report were shown in Table 1 [4, 8].

**Table 1:** Nutritional- and activity status during 3½ days.

Nutritional status during 3½ days (kJ·d^-1^)	Activity status during 3½ days (kJ·d^-1^)
1806.03 ± 517.47	11461.78 ± 734.30

Data are present as mean ± SD of n = 5.

**Fig 1:**
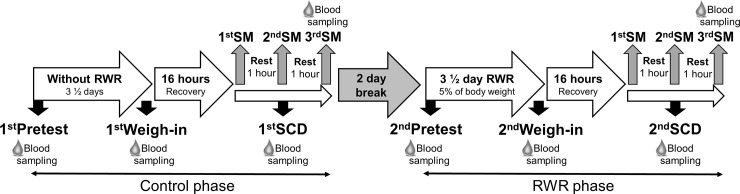
Study design. The study consisted of a control phase without body weight reduction and a rapid weight reduction (RWR) phase. A two day recovery phase was scheduled between control and RWR period. The participants were told to reduce body weight by 5% during 3 ½ days. Each phase consisted of a pretest, weigh-in test after 3½ days and a simulated competition day after 16 h recovery time. On SCD, each athlete executed three TKD simulation matches (SMs) with a 1 h rest between SMs. Blood sampling was conducted at pretest, weigh-in test, SCD and after 3^rd^ SM of both phases.

A two day recovery phase was scheduled between control and RWR period. Each study phase consisted of a pretest, weigh-in test after 3½ days and a competition day after a 16 h recovery time (usual method of weight recovery) (Fig 1). During 16 h recovery, athletes recovered their body water and carbohydrate storage.

On the simulated competition days (SCD), each athlete executed three TKD simulation matches (SMs; 1^st^SM, 2^nd^SM and 3^rd^SM) with a 1 h rest between SMs. According to the guidelines of G1 or G2 (ranking point chart by grade) World Taekwondo (WT) tournaments in Europe, the period of rest between of matches should approximately last 30 min– 2 h. Thus, a mean of resting time of 1 h between matches was chosen in the study. However, these durations depend fully on number of participants (athletes) and organization of tournaments.

In each SM, one test athlete fought against a sparring partner; an opponent who was not a test athlete. The fight displayed real competition conditions: The same athletes fought against each other in both phases (control and RWR).

In accordance with the World Taekwondo rules, each SMs consisted of three rounds and 2 min each, with a resting interval of 1 min in-between. During resting intervals, subjects remained in a seated position (passive). Each match conducted within an official area (8 x 8m). A gas analyzer was placed on the back of the competing athlete and no attack to this area was allowed. The sparring partner remained in a passive position during SM such as blocking and fake motion with the intensive steps. Electronic body protectors and headgear were not used because they would interfere with the spirometry [33–35].

### Anthropometry, hydration, blood sampling and processing

Anthropometric parameters were assessed and venous blood was sampled during pretest (8 am), weigh-in (3 pm) and pre (9 am) as well as post (5 pm) simulated competition day. Anthropometric parameters and hydration status were measured using an 8-electrode segmental multi-frequency bioelectrical impedance analysis (BIA) for body weight (kg) and hydration (%) (seca mBCA 515, GmbH & Co. KG, Germany). mBCA 515 uses four pairs of electrodes which are positioned at each hand and foot, respectively. The 8-electrode technique enables segmental impedance measurement of the arms and legs. Impedance is measured with a current of 100 μA at frequencies between 1 and 1000 kHz [36]. A skinfold Harpenden caliper was used to measure for body fat (%). An experienced investigator measures skin fold at ten sites: cheek, chin, thorax I, triceps, subscapular, thorax II, abdomen, suprailiac, thigh and calf. The mean of duplicate or the median of triplicate measures (when the first two measures differ by more than 5%) was used [37, 38].

Venous blood was sampled from an elbow vein into sodium-heparin tubes (BD Vacutainer, USA) to measure basal blood parameters, RBC deformability and RBC aggregation. Further, blood was sampled into serum vacutainer (BD Vacutainer, USA) to measure glucose and fibrinogen. Capillary blood was additionally sampled from the earlobe into heparinized capillaries (EKF-Diagnostics GmbH, Germany) to measure RBC deformability. Basal blood parameters included RBC (*10^6^·μL^-1^), hemoglobin concentration (Hb) (g·dL^-1^), hematocrit [19] (%), mean corpuscular volume (MCV) (g·dL^-1^), mean cellular hemoglobin [39] (pg) and mean cellular hemoglobin concentration [39] (g·dL^-1^) and was measured using the Sysmex Digitana KX-21N system (Germany).

RBC deformability (venous and capillary blood) and aggregation were analyzed via the Laser-Assisted Optical Rotational Cell Analyzer (LORCA; RR Mechatronics, The Netherlands) [40]. This combined system is an ektacytometer to analyze deformability and syllectometry measurements to determine RBC aggregation. RBC deformability was immediately measured after blood sampling at various fluid shear stresses by laser diffraction analysis at 37°C. The procedure has been described in detailed elsewhere RBCs [8]. Briefly, RBC were mixed with polyvinylpyrrolidone in a 1:250 ratio [41] and sheared in a Couette system at 37°C with a laser beam directed through the sheared sample. Shear stresses between 0.3 and 50 Pa were applied and the horizontal and vertical axis of the laser diffraction pattern, a result of the deformed RBC, was used to calculate an elongation index by the LORCA software. Maximum deformability (EI max) and shear rate required for half of maximum deformability (SS1/2) were calculated using the Lineweaver-Burke equation [42].

Aggregation samples were fully oxygenated (Roller Mixer RM5, 36 rpm). Samples were then filled into the Couette system and aggregation was measured at 37°C. During aggregation measurement RBC were first disaggregated which was achieved by applied shear rate of 500s^-1^ for 3 sec. After motor stop, RBC aggregation starts with rouleaux formation in the beginning followed by 3D aggregate formation during the following 120 sec of aggregation measurement. The laser beam transmits through the sample and the reflected light is recorded by two photodiodes and used by the LORCA software to calculate the aggregation index (AI, %). A final iteration protocol was applied to analyze the threshold shear rate balancing RBC aggregation and disaggregation. The estimated dIsc min is determined as the minimum change in backscatter intensity found during the iteration procedure enabling disaggregation with γ at dIsc min (1/sec) being the shear rate at which dIsc min was detected.

Glucose concentration was analyzed by a photometric system Cobas Mira Plus (Roche Diagnostic, Switzerland). Fibrinogen concentration (ng·mL^-1^) was measured using the Fibrinogen ELISA (Abcam, UK) according to the manufacturer`s guidelines.

During all SMs, heart rate [43], blood lactate concentration (mmol·L^-1^) and oxygen uptake (VO_2_) were additionally monitored. Capillary blood was sampled from the earlobe before and after each SM as well as from the first to seventh minute after last SM [35] to measure lactate concentration (mmol·L^-1^). Lactate concentration (Peak La^-^) was analyzed by an enzymatic-amperometric sensor chip system (Biosen S-line, EKF diagnostic sales, GmbH, Germany).

A video camera (JVC GC-PX10 HD) was used to record the match for following analysis (i.e., to quantify the amount of time spend fighting during the SM). A Polar Pro Team dock (Polar Electro OY, Finland) was used to record heart rate. The mean heart rate (HR_mean_) was calculated as average of the heart rate during the entire round (each SM), while peak heart rate (HR_peak_) was estimated as the highest value during all rounds of each SM [35, 44, 45]. The oxygen uptake in SM and during rest was measured breath-by-breath using the Oxycon Mobile (CareFusion, Germany). The portable spirometric device measures the oxygen concentration through an electrochemical sensor and data are telemetrically transmitted and recorded on a personal computer [46]. The gas analyzer was calibrated using calibration gas (16% O_2_, 5% CO_2_; RiessnerGase, Germany), and the turbine volume transducer was calibrated using a 3 L syringe (Hans Rudolph Inc., USA).

Data were recorded until 6 min after the end of the SM. The mean oxygen uptake (VO_2mean_) was calculated as the average VO_2_ of each SM and the peak oxygen uptake (VO_2peak_) was determined as highest value of each SM.

### Calculation of energy contribution and video analysis

Evaluations of aerobic, anaerobic-lactic and anaerobic-alactic system were conducted by the measurement of oxygen uptake during activity, peak blood lactate concentration, and the fast phase of excess oxygen uptake after exercise (EPOC_FAST_), respectively [35].

Aerobic energy (W_AER_) was calculated by subtracting rest oxygen uptake (VO_2REST_) from VO_2_ during a round by the trapezoidal method in which the area under the curve is divided into pieces and then the sum of each trapezoid is calculated to estimate the integral [35, 47, 48]. VO_2REST_ was determined in the standing position, with the last 30 s of a 5 min period used as a reference. The anaerobic-alactic system (W_PCR_) was calculated considering the oxygen uptake during the interval between rounds, and the fast component of excess post-exercise (EPOC_FAST_) after the third round [33, 47–49]. In this study, the kinetics of post-match oxygen consumption was fitted by mono- and bi-exponential models. The slow component of the bi-exponential model was negligible. Hence, the post-match VO_2_ data was fitted to a mono-exponential model and W_PCR_ was obtained by calculating the integral of the exponential part [33, 47–49].

The contribution of the anaerobic-lactic system (W_La_^-^) was estimated as the lactate concentration after combat, assuming that the accumulation of 1 mmol·L^-1^ (La^-^) is equivalent to 3ml O_2_ kg^-1^ of body mass [50]. The caloric quotient of 20.92 kJ [51] was used in all three different energy systems. The difference of lactate (ΔLa^-^) was calculated as the lactate concentration after the round, minus the lactate concentration at the beginning of the round. Total energy demand (W_TOTAL_) was calculated as the sum of the three energy systems (W_AER_ + W_PCR_ + W_La_^-^) in kJ [35].

According to the criteria of Santos et al. [52], the recorded matches were analyzed using the Dartfish Version 7 software. Time-motion analyzes were conducted in an attempt to calculate work/rest ratios for each match. The events were measured in seconds and tenths of seconds, using the marking tool in the analysis software. An attack was considered the time from the beginning of a foot or hand movement in the direction of the opponent up to the athlete stopped his attack movement or unable to continue attack (because of a fall or referee pause). Step movement was also determined as the entire phase between attacks when there was no break in the match. Pause was determined as the stops in SM ordered by the referee. Step time was added to the referee pause-times to determine the phase without attack (PWA), together with the stopwatch stop-time called out by the referee. Attack was considered the total time during which one of the athletes tried to attack or succeeded in attacking his opponent [35]. For the ratio between High Intensity Movement (HIM) and Low Intensity Movement (LIM), sum of time without attacks was divided by the number of attacks ratio [35]. The analyses showed a high intra-class correlation [53] of 0.93 which corresponds to other studies [35, 54, 55].

### Statistical analysis

Statistical software GraphPad Prism 6 (LaJolla, USA) was used for the statistical analyses of the data and graphical representation of data. The data are presented as means ± SD. The mean value of SM for performance parameters and energy demands was calculated by all 3 rounds of each SM and statistically compared. The variables of each phase (control and RWR) were compared using a Friedman repeated-measures rank test with post-hoc Dunn`s. The same measurement time e.g., 1^st^SM (control) vs.1^st^SM (RWR) was analyzed using a Wilcoxon signed ranks test. Statistical difference was considered to be significant for P<0.05 and P<0.01. The effect size (r = Z/√𝑁) was calculated for the non-parametric tests [56]. Whiskers box plot present minimum to maximum and median values. Thresholds for small, medium, and large effects were 0.10, 0.30, and 0.50, respectively [57]. Additionally, a Pearson’s two-tailed correlation and linear regression analyses were performed between kick frequency (KF) and VO_2peak_, VO_2mean_, peak La^-^ and ΔLa^-^.

## Results

### Anthropometric data, hydration and glucose

**Fig 2:**
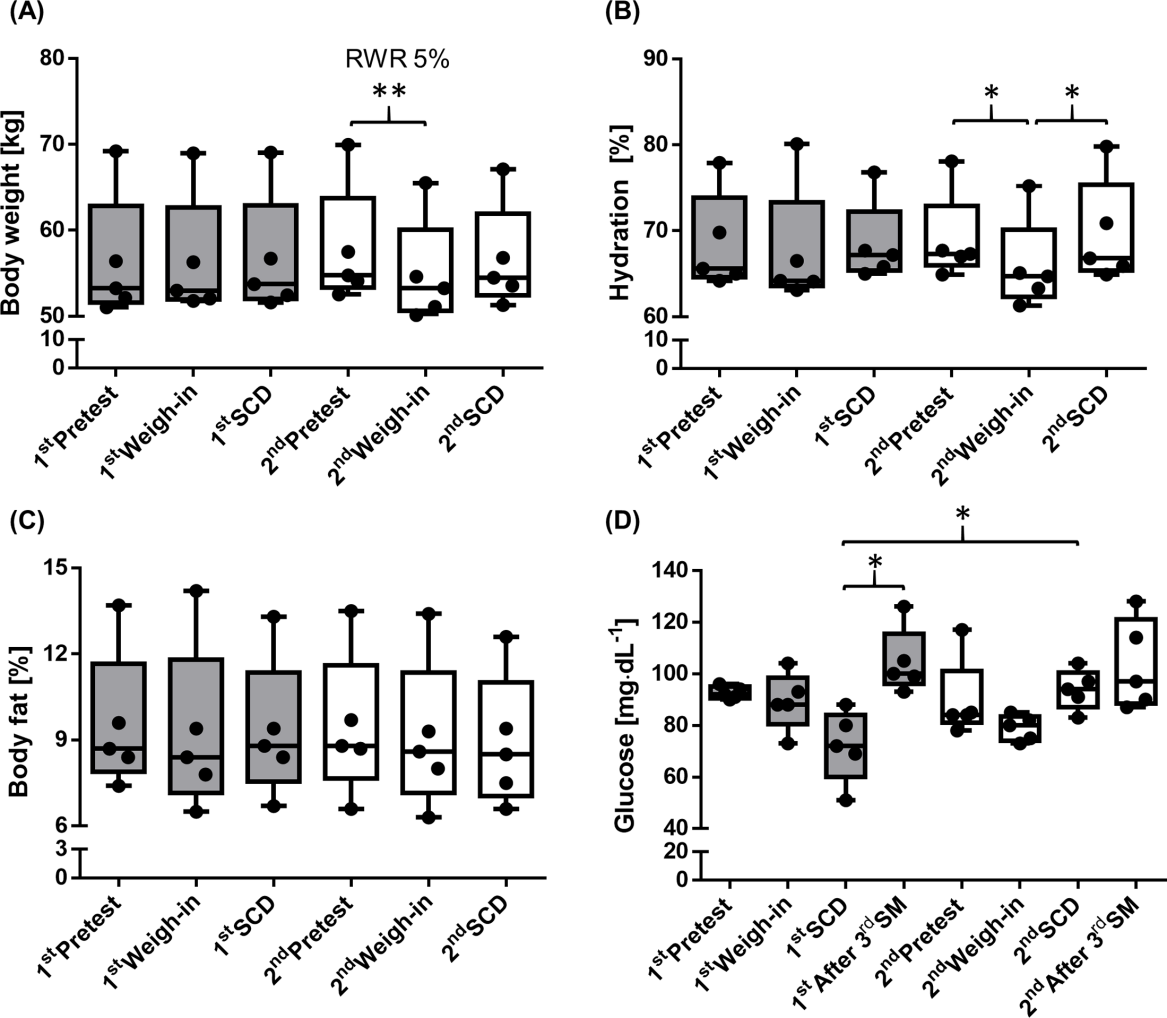
Anthropometric data, hydration and glucose. (A) Body weight significantly decreased at weigh-in of RWR phase (P = 0.003). (B) Hydration status was significantly decreased during weigh-in compared to pretest and increased on SCD (P = 0.021, P = 0.035, respectively) of RWR phase. (C) Body fat remained unaffected by the interventions. (D) Serum glucose concentration was significantly increased after 3^rd^SM of control phase (P = 0.003) and a significant difference was detected between SCD of control and RWR phase (P = 0.031). All data are presented as box plot (n = 5).

In RWR, athletes significantly reduced body weight between 2^nd^pretest und 2^nd^weigh-in (P = 0.003; r:-0.63). Hydration status was significantly decreased in 2^nd^weigh-in compared to 2^nd^pretest and again increased on 2^nd^SCD compared to 2^nd^weigh-in (P = 0.021; r:-0.63, P = 0.035; r:-0.63, respectively). Body weight was unaltered in the control phase. Body fat was not affected by the interventions. Serum glucose concentration showed a significant increase after 3^rd^SM compared to SCD of control phase (P = 0.003; r:-0.63) and another significant difference on SCD between control and RWR (P = 0.031; r:-0.63) (Fig 2A–2C).

### Basal blood parameters

Basal blood parameters are presented in Table 2. The hematocrit concentration was significantly lower at 2^nd^pretest compared to 1^st^pretest (P = 0.032; r = -0.64). Other basal blood parameters were not affected by the interventions.

**Table 2:** Basal blood parameters of all measurements.

	1^st^Pretest	1^st^Weigh-in	1^st^SCD	1^st^After 3^rd^SM	2^nd^Pretest	2^nd^Weigh-in	2^nd^SCD	2^nd^After 3^rd^SM
RBC (*106·μL^-1^)	5.89 ± 0.26	5.28 ± 0.14	5.39 ± 0.26	5.28 ± 0.28	5.08 ± 0.20	5.68 ± 0.44	5.03 ± 0.16	5.07 ± 0.14
Hb (g·dL^-1^)	17.96 ± 0.75	15.62 ± 0.91	15.72 ± 0.46	15.32 ± 1.48	14.76 ± 0.50	16.56 ± 2.11	14.96 ± 1.15	15.30 ± 0.97
Hct (%)	50.30 ±1.83	44.94 ± 2.30	46.00 ± 0.74	45.06 ± 2.83	43.58 ± 1.60 ^a^*	46.50 ± 6.96	43.12 ± 2.85	43.40 ± 2.18
MCV (fl)	85.52 ± 3.03	85.02 ± 3.27	85.40 ± 3.12	85.38 ± 3.40	85.78 ± 3.02	84.78 ± 3.37	85.70 ± 3.14	85.66 ± 3.29
MCH (pg)	30.58 ± 1.83	29.56 ± 1.25	29.22 ± 1.86	28.98 ±1.97	29.08 ±1.63	30.42 ± 1.76	29.72 ± 1.58	30.20 ± 2.02
MCHC (g·dL^-1^)	35.72 ± 1.12	34.76 ± 0.99	34.16 ± 1.38	33.94 ±1.36	33.88 ± 0.99	35.76 ± 1.75	34.68 ± 1.49	35.28 ± 1.89

Data are present as mean ± SD of n = 5.

RBC: red blood cell; PLT: platelet; Hb: hemoglobin; Hct: hematocrit; MCV: mean corpuscular volume; MCH: mean corpuscular hemoglobin; MCHC: mean corpuscular hemoglobin concentration; SCD: simulated competition day; SM: simulation match.

a: 1^st^Pretest

*P<0.05

### Hemorheological properties

**Fig 3:**
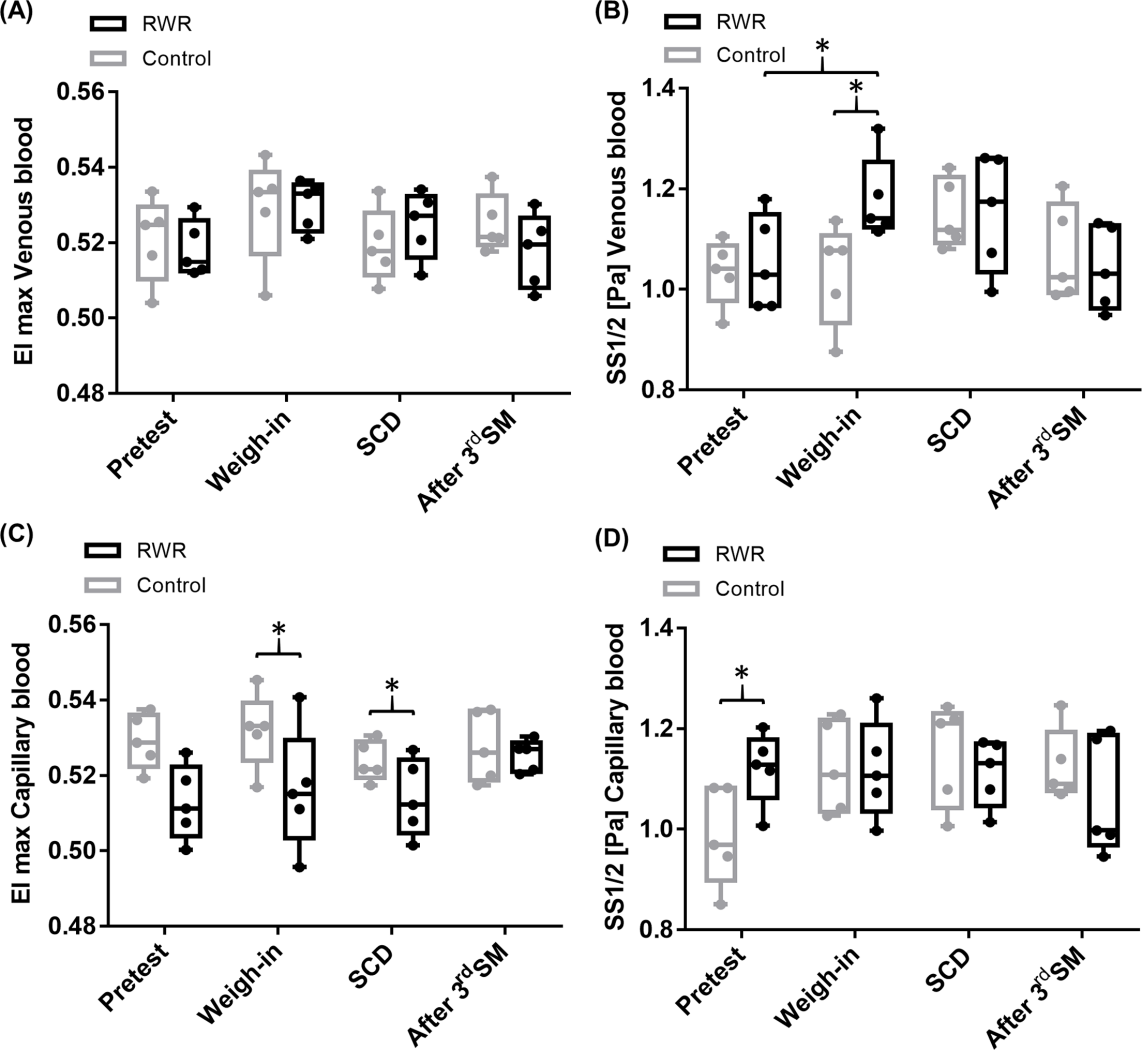
RBC maximum deformability (EI max) and shear rate for half of maximum deformability SS1/2 (Pa). (A) EI max of venous blood showed no significant difference during interventions nor between set-ups. (B) During RWR, SS1/2 of venous blood significantly increased at weigh-in with significantly higher values compared to pretest of control phase (P = 0.019; P = 0.031, respectively). (C) EI max of capillary blood was significantly higher at weigh-in and SM in RWR compared to control phase (P = 0.031). (D) SS1/2 of capillary blood was significantly lower at pretest in control phase compared to RWR (P = 0.031). All data are presented as box plot (n = 5).

Calculated EI max of venous blood showed no differences between the two set-ups (control/RWR) and no alteration during the interventions. In RWR, SS1/2 (Pa) significantly increased from pretest to weigh-in. Values were also significantly higher at weigh-in compared to weigh-in of control phase (P = 0.019; r:-0.63, P = 0.031; r:-0.63, respectively). EI max values of capillary blood were higher in control phase at weigh-in and SCD compared to RWR (P = 0.031; r:-0.63, respectively). In RWR, SS1/2 of capillary blood was significantly higher at pretest compared to control pretest (P = 0.031; r:-0.63) (Fig 3A–3D).

**Fig 4:**
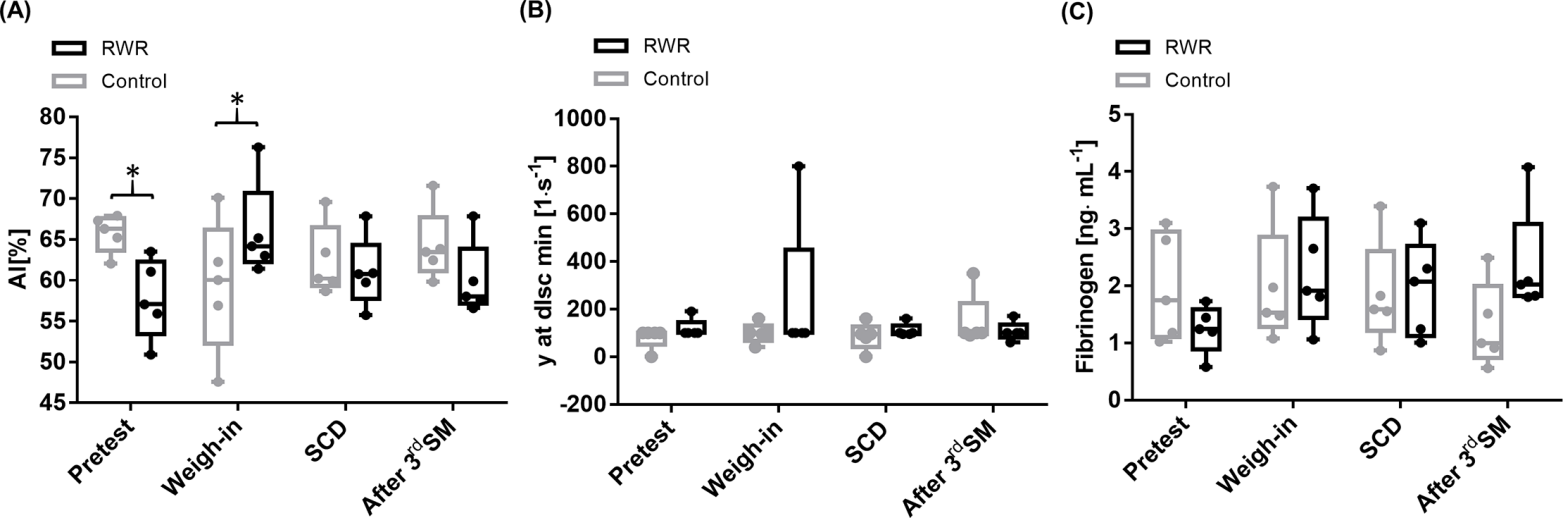
Aggregation index (AI, %), disaggregation threshold (γ at dISCmin, 1·s^-1^) and fibrinogen concentration. (A) AI showed significant differences in pretest and weigh-in between control and RWR (P = 0.003, P = 0.031, respectively). (B) Disaggregation threshold and (C) fibrinogen concentration were not affected by the interventions. All data are presented as box plot (n = 5).

AI (%) was significantly higher at pretest in control phase compared to RWR (P = 0.031; r:-0.63). AI was significantly higher at weigh-in of RWR compared to control (P = 0.031; r = 0.63). The disaggregation threshold showed no significant difference during the interventions and between set-ups. Serum fibrinogen concentration was not affected by the interventions (Fig 4A–4C).

### Physiological parameters

HR_mean_ significantly increased during 3^rd^SM of RWR compered to 3^rd^SM control (P = 0.031; r:-0.63). HR_peak_ was significantly higher in 3^rd^SM of RWR compared to 2^nd^SM of RWR (P = 0.012; r:-0.64). VO_2mean_ was significantly higher at 3^rd^SM of RWR compared to control (P = 0.047; r:-0.63). VO_2peak_ and absolute VO_2peak_ were significantly higher at 2^nd^SM and 3^rd^SM of RWR compared to control (P = 0.031; r:-0.64, respectively). Peak La^-^ und ΔLa^-^ were significantly decreased in 3^rd^SM compared to 1^st^SM of control (P = 0.006; r:-0.64, P = 0.002; r:-0.63, respectively) and significantly lower compared to corresponding values of RWR (P = 0.031; r:-0.63, P = 0.021; r:-0.63, respectively) (Table 3). Kick frequency (KF) was significant different between control and RWR in 1^st^SM and 3^rd^SM (P = 0.031; r:-0.64, P = 0.031; r:-0.63, respectively) (Table 4).

**Table 3:** Physiological parameters and calculated energy demands of Taekwondo simulation match during control and rapid weight reduction phases.

	1^st^SM (control)	2^nd^SM (control)	3^rd^SM (control)	1^st^SM (RWR)	2^nd^SM (RWR)	3^rd^SM (RWR)
HR_mean_ (beats·min^-1^)	181.8 ± 8.2	182.8 ± 6.5	178.8 ± 8.2	179.6 ± 10.9	176.8 ± 8.7	185.8 ± 3.2^c^^*^
HR_peak_ (beats·min^-1^)	192.4 ± 5.8	190.6 ± 4.4	193.6 ± 8.3	193.8 ± 8.8	188.6 ± 5.4	196.2 ± 5.0^e^^*^
VO_2mean_ (mL·kg^-1^·min^-1^)	34.2 ± 2.9	35.5 ± 1.7	33.2 ± 2.0	34.9 ± 2.2	37.3 ± 1.9	38.7 ± 3.0^c^^*^
VO_2peak_ (mL·kg^-1^·min^-1^)	40.0 ± 2.9	40.9 ± 2.9	40.8 ± 2.0	40.8 ± 3.3	44.0 ± 2.7^b^^*^	46.7 ± 6.1^c^^*^
VO_2peak_ (mL·min^-1^)	2259.0 ± 252.3	2315.3 ± 219.0	2327.1 ± 295.9	2304.3 ± 199.3	2487.0 ± 221.9^b^^*^	2632.1 ± 223.7^c^^*^
Peak La^-^ (mmol·L^-1^)	9.2 ± 3.9	7.2 ± 4.2	5.6 ± 4.2^a^^**^	8.2 ± 4.4	7.4 ± 3.7	8.1 ± 2.9^c^^*^
ΔLa^-^ (mmol·L^-1^)	2.7 ± 1.1	2.0 ± 1.2	1.5 ± 1.0^a^^**^	2.2 ± 1.5	2.0 ± 0.8	2.3 ± 0.9^c^^*^
W_PCR_ (kJ)	18.8 ± 3.5	19.9 ± 3.3	19.5 ± 2.3	21.3 ± 5.5	21.0 ± 5.5	22.2 ± 4.9
W_PCR_ (%)	19 ± 3	20 ± 4	21 ± 3	21 ± 6	20 ± 6	20 ± 4
W_La_^-^ (kJ)	10.1 ± 5.5	7.7 ± 5.9	5.6 ± 4.7 ^a^^**^	8.3 ± 6.6	7.2 3.9	8.6 ± 4.2^c^^*^
W_La_^-^ (%)	9 ± 4	7 ± 4	5 ± 4 ^a^^*^	8 ± 5	6 ± 3	8 ± 3 ^c^^*^
W_AER_ (kJ)	72.8 ± 10.4	75.3 ± 7.5	71.9 ± 12.5	73.9 ± 9.1	79.6 ± 8.5 ^b^^*^	82.4 ± 7.2
W_AER_ (%)	72 ± 3	73 ± 2	74 ± 2	71 ± 1	74 ± 4	72 ± 2
W_TOTAL_ (kJ)	101.7 ± 16.1	102.5 ± 11.6	97.0 ± 17.9	103.5 ± 12.9	107.8 ± 8.5	113.2 ± 8.4

Data are present as mean ± SD of n = 5.

RWR: phase of rapid weight reduction, SM: simulation match, HR_mean_: mean heart rate, HR_peak_: highest heart rate, VO_2mean_: mean oxygen uptake, VO_2peak_: highest oxygen uptake, Peak La^-^: highest lactate concentration, ΔLa^-^: difference of lactate concentration after the round minus at the beginning of the round, W_PCR_: anaerobic-alactic demand, W_La_^-^: anaerobic-lactic demand, W_AER_: aerobic demand.

a: first simulation match of control

b: second simulation match of control

c: third simulation match of control

e: second simulation match of rapid weight reduction phase

*P<0.05

**P<0.01

**Table 4:** Kick frequency, attack time, total time of attacks, total time without attacks, ratio between attacks and time without attacks, and ratio between total time without attacks and number of attacks (HIM vs. LIM).

	1^st^SM (control)	2^nd^SM (control)	3^rd^SM (control)	1^st^SM (RWR)	2^nd^SM (RWR)	3^rd^SM (RWR)
Kick frequency (number of attacks)	15.73 ± 5.83	16.87 ± 4.16	16.53 ± 3.44	20.73 ± 5.10^a^^*^	20.11 ± 6.56	21.73 ± 6.31^c^^*^
Attack time (s)	0.87 ± 0.04	0.86 ± 0.05	0.86 ± 0.06	0.84 ± 0.05	0.85 ± 0.06	0.86 ± 0.07
Sum of attacks time (s)	14.07 ± 5.99	15.40 ± 5.68	13.80 ± 3.63	17.60 ± 5.18	18.00 ± 7.97	18.80 ± 6.53
Sum of time without attacks (s)	106.13 ± 5.95	104.67 ± 5.54	106.27 ± 3.61	102.53 ± 5.28	101.93 ± 7.91	101.20 ± 6.50
Attacks/without attacks ratio	0.27 ± 0.37	0.15 ± 0.06	0.13 ± 0.04	0.17 ± 0.07	0.18 ± 0.10	0.19 ± 0.08
Sum of time without attacks/number of attacks ratio	8 ± 3	7 ± 2	7 ± 2	5 ± 2	6 ± 2	5 ± 2
HIM vs. LIM	1:8	1:7	1:7	1:5	1:6	1:5

Data are present as mean ± S.D. of n = 5.

RWR: phase of rapid weight reduction, SM: simulation match, HIM: high intensity movement; LIM: low intensity movement.

a: first simulation match of control

c: third simulation match of control

*P<0.05

**Fig 5:**
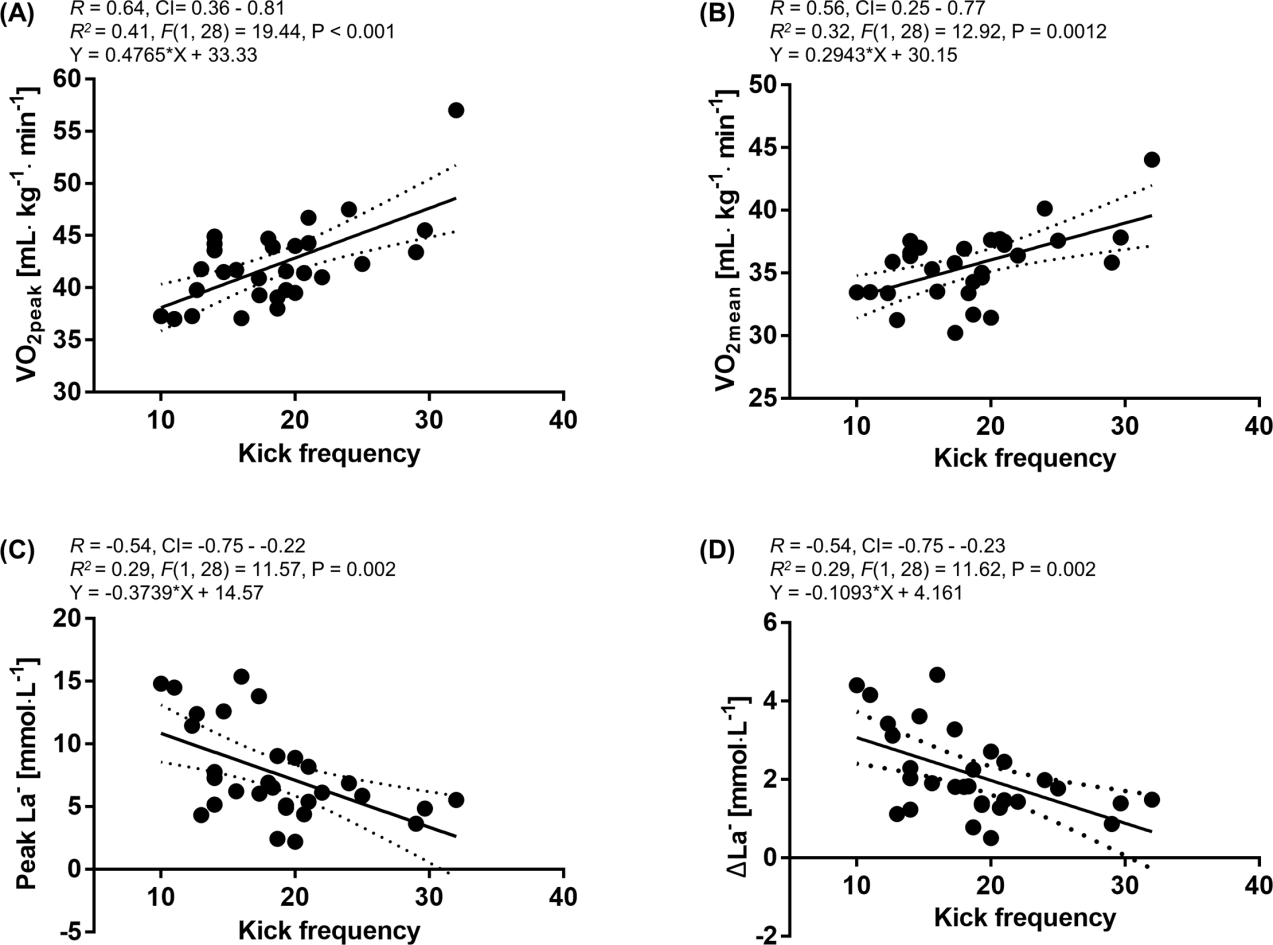
Pearson’s two-tailed correlation and linear regression between kick frequency (KF) and peak oxygen uptake (VO_2peak_), mean oxygen uptake (VO_2mean_), peak lactate (peak La^-^) and difference of lactate ΔLa^-^. (A) Moderate positive correlation and linear regression between KF and VO_2peak_ (B) Moderate positive correlation and linear regression between KF and VO_2mean_ (C) Moderate negative correlation and linear regression between KF and peak ΔLa^-^ (D) Moderate negative correlation and linear regression between KF and peak La^-^.

Moderate positive correlations and linear regressions were observed between VO_2peak_, VO_2mean_ and KF (r = 0.64; 95%CI: 0.36–0.81; R^2^ = 0.41; *F* = 19.44; P<0.001, r = 0.56; 95%CI: 0.25–0.77; R^2^ = 0.32; *F* = 12.92; P = 0.0012, respectively). Moderate negative correlations and linear regressions were calculated for peak La^-^, ΔLa^-^ and KF (r = 0.54; 95%CI: -0.75 - -0.22; R^2^ = 0.29; *F* = 11.57; P = 0.002, r = 0.54; 95%CI: -0.75 - -0.23; R^2^ = 0.29; *F* = 11.62; P = 0.002, respectively). (Fig 5A–5D).

### Energy demands during SM

W_TOTAL_ and W_PCR_ showed no significant difference during interventions and no difference between set-ups. W_La_^-^ was significantly lower during 3^rd^SM compared to 1^st^SM of control (P = 0.002; r:-0.63, P = 0.005; r:-0.64, respectively), and significantly higher values were measured during 3^rd^SM of RWR compared to control (P = 0.021; r:-0.63, P = 0.031; r:-0.63, respectively). W_AER_ was significantly higher during 2^nd^SM of RWR compared to 2^nd^SM of control (P = 0.031; r:-0.63) (Table 3).

The technical actions and the time spent on each SM are described in Table 4. Kick frequency, attack time, sum of attack time, sum without attack time, attacks/without attacks ratio and sum of time without attacks/number of attacks ratio were identified.

## Discussion

The effect of rapid weight reduction on TKD-specific performance parameters after a practically relevant recovery time has not been investigated so far. Thus, to our knowledge this is the first study investigating the effects of a 16 h recovery time after weight reduction by 5% within 3½ days on physiological and hemorheological responses and energy demands with special regards to multiple TKD SMs. The major findings indicate that RWR impairs hemorheological but not hematological parameters. The values returned to pretest level after the 16 h recovery period. Performance related parameters such as VO_2peak_ were higher during SMs after RWR, most likely because of higher kick frequency during this phase. Hematological parameters were monitored during RWR and SMs and compared to a control phase with no weight reduction. Basal blood parameters included RBC count, hematocrit, hemoglobin concentration, MCV, MCH and MCHC. Blood parameters give some indication of hydration status and blood viscosity. MCHC for example was described to sensitively reflect cell viscosity within RBC [39] and increased hematocrit was shown to negatively influence blood viscosity. Alterations of these parameters were associated with a reduction in performance capacity because increased viscosity reduces peripheral blood flow and cardiac output [21, 58, 59]. Increases in athletes`hematocrit may be caused by extensive loss of body water during training or fasting [60]. Here, MCH(C) and hematocrit were not affected by the interventions, although hydration status was significantly decreased after RWR. Several studies indicate that hematocrit does not necessarily increase loss of body water. Loss of body water < 3% of body mass does not affect the plasma volume [7, 8, 61–63]. However, hematocrit was significantly higher at 1^st^pretest compared to 2^nd^pretest. Hematocrit is affected by a variety of factors including hydration status and abovementioned mechanisms. This might have influenced the values. Inaccuracies of measurement might also explain the high hematocrit results and might thus represent a possible limitation of the study.

Besides MCH(C) and hematocrit, other blood values were also not affected by the intervention. In regard of RBC deformability, MCV is of special interest. Both, increased and decreased MCV values were described to alter RBC deformability [64]. The values described for this study were not affected by RWR or altered upon SMs and were all within normal range (80–98 fl) [65]. To evaluate the effects of RWR, recovery phase and SMs on RBC deformability, EI max was measured in capillary (arterial-venous mix blood) and venous blood, respectively. EI max of capillary and of venous blood remained unaltered during RWR, respectively, and results thus in part reflect our recent findings [8]. Values of capillary blood were in general higher in control compared to RWR with significant differences at weigh-in before SMs but this difference might not be related to the interventions.

EI max represents a theoretical values and might not be sufficient to state the effect of RWR on deformability. Thus, shear rate necessary for one half of maximum deformability (SS1/2) was calculated. In venous blood, SS1/2 significantly increased after RWR indicating that more shear stress is necessary to deform RBC and thus indicates reduced deformability. In capillary blood, no such behavior was measured. These differences between capillary and venous blood might be caused by differing flow resistance in vascular systems [66]. A decrease in RBC deformability might also be caused by food restriction during RWR. Several studies associated a reduction in RBC deformability to decrease in blood glucose and thus reduced ATP levels [8, 67, 68]. Glucose levels were reduced on weigh-in of RWR and might in part relate to the reduced RBC deformability. A recent study indicated that food restriction and reduced glucose levels reduce nitric oxide production in RBC which in turn reduces RBC deformability [8]. It remains to be investigated whether RWR facilitates oxidative/nitrosative stress which was shown to reduce RBC deformability [69].

RBC aggregation just like RBC deformability define blood flow in the microcirculation and might thus be of importance in terms of performance capacity. The results presented herein showed higher RBC aggregation value at weigh-in of RWR compared to the control situation. Further, shear rate balancing aggregation and disaggregation was higher at weigh-in after RWR. Both results are in accordance to recent findings by Yang et al. [8]. Dehydration as monitored for RWR might have caused an increase in blood viscosity thus leading to hyper-aggregation [8, 39, 70].

RBC aggregation is a physiological phenomenon that occurs at the access of the capillaries. RBC aggregation is influence by the plasma protein fibrinogen with increased aggregation behavior at higher fibrinogen levels [71]. RBC aggregation can influence hemodynamics, RBC distribution, and flow dynamics in the microcirculation. Low protein intake through caloric restriction appears to relate to the increase of fibrinogen and aggregation [72]. Hence, serum fibrinogen levels were measured but results suggest that fibrinogen levels were not affected by RWR. Solá et al. [73] and Poggi et al. [74] suggested that other, yet unknown factors, are responsible for an increase in RBC aggregation without a parallel change in fibrinogen levels.

Both, RBC deformability and aggregation values returned to pretest level after a 16 h recovery period and no changes for the parameters were found upon SMs. The data thus suggest that, although RWR might impair hemorheological parameters, recovery time between weigh-in and competition is sufficient to recover blood parameters. In case of reduced performance capacity on competition day, it seems unlikely that this might be associated to impaired hemorhological responses. Nevertheless, it remains to be investigated whether repeated RWR applications might have a bigger effect.

TKD-specific performance parameters were monitored during SMs. VO_2mean_, VO_2peak_, absolute VO_2peak_ and aerobic demand were higher in 2^nd^SM and 3^rd^SM of RWR compared to control. Accordingly, HR_mean_ and HR_peak_ were increased in 3^rd^SM of RWR compared to 3^rd^SM of control. This might be related to increased kick frequency measured during SMs of the RWR phase as also indicated by lactate measurements [35, 45]. Other studies confirmed that RWR does not impact performance (determined in wrestlers and boxer) after a recovery period of 3–20 h [3, 75].

Metabolic systems were measured during SMs. The aerobic system was found to dominate during all simulated matches. The results might be explained by frequent low intensity movement which includes the pauses and step movements [35, 52, 76, 77]. Furthermore, the ratio of high intensity to low intensity movement (HIM to LIM) was from 1:5 to 1:8.

**Fig 6:**
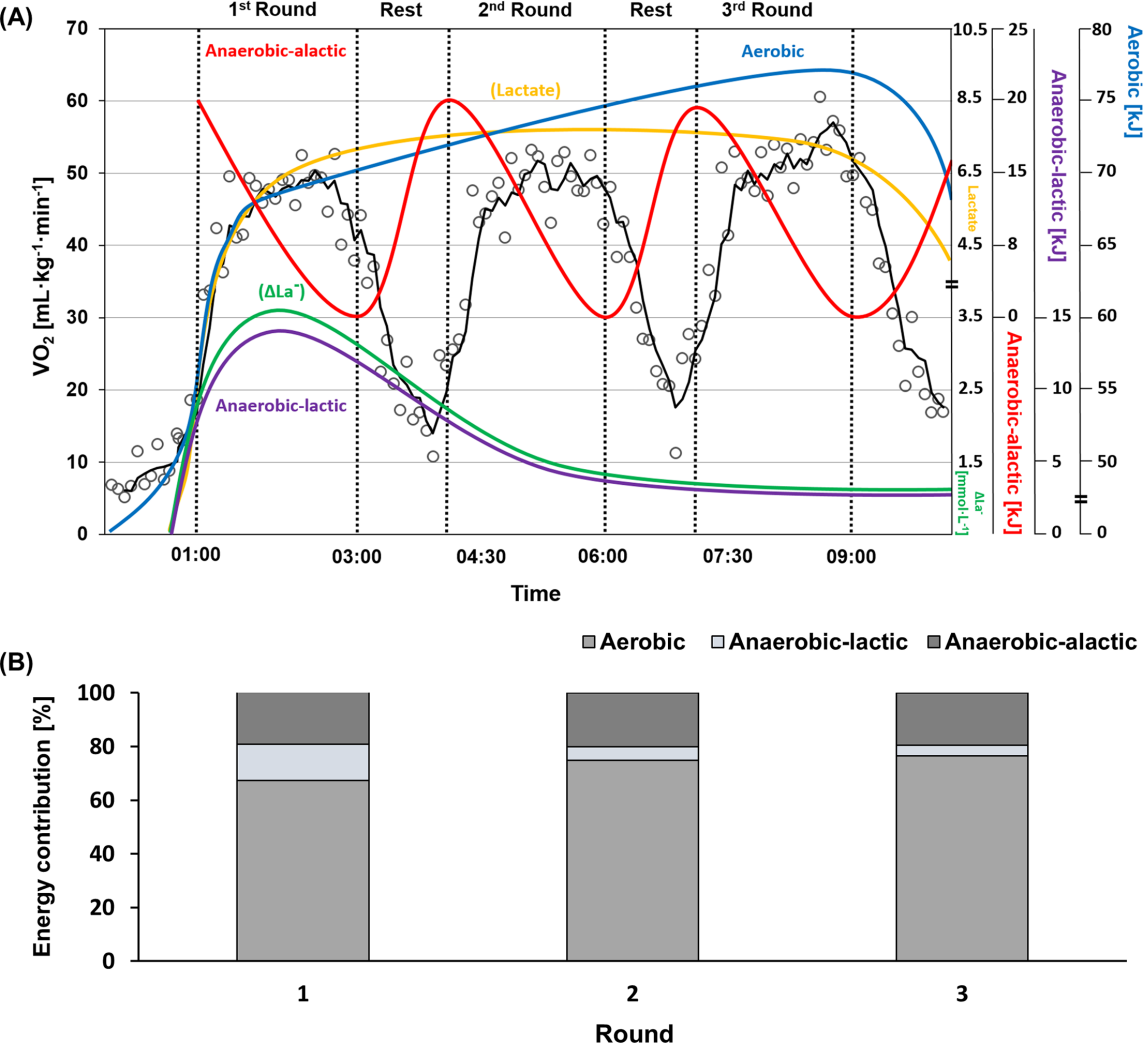
**Averages of each energy system during all TKD simulation matches (30 matches) in kJ (A) and % (B).** Black: An example of VO_2_ (mL·kg^-1^·min^-1^) during a simulation match, red: Anaerobic-alactic demand, violet: Anaerobic-lactic demand, blue: aerobic demand (kJ), yellow: lactate, green: difference of lactate (mmol·L^-1^). All illustration of energy systems and lactate values based on mean values of each parameters.

Anaerobic-alactic system showed no alteration in both phases. Recovery interval during pauses and rounds with LIM might be sufficient for the recovery of phosphocreatine, which indicated no alteration between control and RWR phases [35, 78, 79]. La^-^, ΔLa^-^ and anaerobic-lactic demand were also higher in RWR than those in control because more HIM was done in RWR. Indeed, our regression analyses showed moderate positive and negative relationships between these parameters. The results are thus in accordance to a computer simulation program which indicates that the aerobic system is used during moderate and increasing duration of exercise while anaerobic-lactic system is less required (Fig 6) [80, 81]. Similar results were reported by Campos et al. [35] showing that the aerobic system (66%) was predominantly used during TKD simulation matches.

To avoid an impact of RWR on performance parameters, TKD athletes delimitate body weight reduction to 5% in praxis. And to protect athletes health situation, World Taekwondo should consider a random control of body weight in the morning of the competition day to ensure no discrepancy of body weight between weigh-in and competition day.

## Conclusion

The findings of the present pilot study indicated that RWR by 5% body weight does not impair physiological and hemorheological parameters on the simulated competition when a 16 h recovery time was scheduled between weigh-in and competition day. However, it remains to be investigated whether repeated RWRs lead to different results in the season.

## Supporting information

S1 Checklist TREND statement checklist. (PDF)
